# Influence of Diode Laser for the Treatment of Dentin Hypersensitivity on Microleakage of Cervical Restorations

**DOI:** 10.1155/2021/9984499

**Published:** 2021-04-30

**Authors:** Doaa R. M. Ahmed, Diana G. Shaath, Jomana B. Alakeel, Abdulaziz A. Samran

**Affiliations:** ^1^Conservative Dentistry Department, Faculty of Dentistry, Alexandria University, Alexandria, Egypt; ^2^BDS, College of Dentistry, Dar Al Uloom University, Riyadh, Saudi Arabia; ^3^Department of Restorative and Prosthetic Dental Sciences, College of Dentistry, Dar Al Uloom University, Riyadh, Saudi Arabia; ^4^College of Dentistry, Ibb University, Ibb, Yemen

## Abstract

Noncarious cervical lesions (NCCLs) are a common clinical finding often linked with dentin hypersensitivity (DH). *Aim*. The aim of the study was to evaluate the influence of diode laser for the treatment of DH on microleakage of subsequent NCCL restorations. *Materials and Methods*. Forty-eight extracted human premolars were collected. All teeth received standardized cervical preparation on both the buccal and palatal surfaces and were randomly divided into three groups (*n* = 16) according to the restorative material used: nanohybrid composite resin (CR), resin-modified glass ionomer (RMGI), and conventional glass ionomer (GIC). The prepared cavities on the palatal surfaces were treated by diode laser using SIROlaser Blue (Sirona Dental Systems, Bensheim, Germany) prior to restoration, while preparations on the buccal surfaces were directly restored. After thermocycling, the teeth were immersed in methylene blue dye for microleakage evaluation under 40x magnification at both occlusal and cervical margins. The Kruskal-Wallis test followed by the Bonferroni tests was conducted to determine inter- and intragroup differences (*P* < 0.05). *Results*. All restorative materials tested showed some degree of microleakage with no statistically significantly different scores with or without the use of laser desensitization prior to restorative treatment. Group CR showed the least microleakage, followed by group RMGI, while group GIC showed the highest. Cervical margins showed greater microleakage than the occlusal margins where the difference was statistically significant in the RMGI group without laser pretreatment (*P* = 0.006) and in both groups CR (*P* = 0.02) and RMGI (*P* = 0.006) with the laser pretreatment. *Conclusion*. Application of diode laser for the treatment of DH prior to the restoration of teeth with NCCL did not affect the microleakage of all the restorative materials tested. All the materials showed some degree of microleakage, which was higher in gingival margins compared to occlusal margins. The resin composite shows the least microleakage among all the tested materials.

## 1. Introduction

Noncarious cervical lesions (NCCLs) are defined as tooth structure loss at or near the cementoenamel junction of the teeth that is not associated with the presence of caries [1]. The etiology of NCCLs is considered multifactorial, where the lesions result from the interaction of different mechanisms including biocorrosion, mechanical stress, and friction [2]. The prevalence of these lesions is variable, reaching as high as 85% as reported by certain studies, and was shown to be closely associated with people's lifestyles [3]. Parafunctional habits, inadequate tooth brushing, high acidic beverage intake [2], and even certain athletic activities are considered among the risk factors [1]. Maxillary posterior teeth especially premolars are the most susceptible teeth [4, 5] possibly due to their smaller crown size and their location in the dental arch, exposing them to excessive lateral forces during mandible excursive movements thus leading to amplified deformations at the cervical areas [6, 7].

NCCLs usually worsen with age and appear as wedge or saucer-shaped lesions of variable depth and width [8, 9] depending on the duration, intensity, and frequency of the etiologic factors [2]. In addition to being tooth defects possibly allowing for plaque accumulation with resulting biological hazards on both the tooth structure and the surrounding gingival tissue, NCCLs often extend to expose dentin at the cervical area of the affected teeth, which has been linked with dentin hypersensitivity (DH) [3]. DH is characterized by an acute, short-term pain initiated by different stimulants such as the touch of an instrument, brush bristle, cold and sweet drinks, and those that cannot be ascribed to any other dental defect or pathology [10]. These symptoms are intensified in deeper lesions due to the proximity to the pulp and the greater amount of exposed dentinal tubules [11].

NCCLs associated with DH not only affect esthetics but also the overall quality of life by impacting everyday habits such as speaking, drinking, eating, and tooth brushing [12, 13]. Unfortunately, these increasingly prevalent clinical conditions still pose a significant management challenge for the clinician [14].

Several options have been proposed for the treatment of NCCLs ranging from merely observational treatment to more invasive treatments such as cavity preparation and restoration. According to the minimal intervention dentistry concepts, asymptomatic small noncarious cervical tooth defects may be just observed during follow-up visits. However, it has been highly recommended to restore the teeth when the lesions are of considerable size. Du et al. recommended that NCCLs deeper than 1.5 mm should be restored to prevent further dentin destruction, hypersensitivity, and possible pulpal involvement. The restorations are intended to restore normal tooth morphology and function and to allow for normal stress distribution at the cervical region of the teeth thus preventing further deterioration [15, 16]. The decision of which management approach will be followed is mainly dependent on several patients' factors and the severity of the lesion. When the NCCLs are accompanied by dentin hypersensitivity, relieving patients' discomfort and addressing the issue by dentin desensitization are primarily indicated [10]. This can be achieved using several minimally invasive modalities, classified according to their mechanism of action. The first treatment modality is by aiming to lower nerve sensitivity by blocking synapsis between nerve cells. Nerve desensitizing agents, such as potassium nitrate, mainly incorporated in toothpastes, have been recommended for such use. The other approach is by occluding the exposed dentinal tubules, which can be achieved by different agents, among which are fluoride, glutaraldehyde, casein phosphopeptide/amorphous calcium phosphate (CPP/ACP), nanohydroxyapatite, and bioactive glass.

In recent years, the use of lasers has become increasingly common in treating cervical dentin hypersensitivity [17]. The lasers interact with tooth tissue, causing different reactions depending on the wavelength and the power density of the laser source, as well as the optical characteristics of the target tissue. Medium-power lasers such as CO_2_ and Er: YAG lasers were shown to decrease sensitivity by occluding the dentinal tubules, while low-power output lasers (such as diode lasers) are assumed to mediate analgesic effects by depressing neural conduction. This effect is believed to be achieved by blocking C-fiber afferent depolarization [18]. Nevertheless, when treatment of dentin hypersensitivity is not successful and when the lesion size could jeopardize tooth vitality, structural integrity, esthetics, or periodontal health, restorative treatments are required. Various material options are available for restoring NCCLs, such as glass ionomer cement (GIC), resin-modified glass ionomer (RMGI), and composite resin (CR), with variable success rates [5, 19]. CR has favorable esthetics and mechanical properties yet a higher modulus of elasticity and polymerization shrinkage, risking the quality of the bond to the tooth structure. GIC, on the other hand, has a modulus of elasticity similar to that of dentin, bonds chemically to enamel and dentin, and releases fluoride but is inferior to CR in terms of esthetic properties. RMGI was developed to combine the advantages of both CR and GIC by adding the functional monomers of photopolymerizable resins to GIC [8].

The difficulty in isolation, the geometric shape of the lesions, the characteristics of the dentinal substrate available for bonding, and the properties of the restorative materials all present challenges that could affect the restorations' success. One of the main reasons for the failure of NCCL restorations is microleakage, a process of gap formation at the interface between the tooth surface and the restoration. These interfacial defects could develop as a result of the physical and chemical properties of the restorative materials, as well as from exposure of the restored teeth to thermal and mechanical stresses. The consequences of such defects can in turn lead to increased hypersensitivity, staining at the restorations' margins, recurrent caries, and possibly pulpal involvement [20].

Moreover, it has been observed that DH may continue even after restorative treatment is completed, especially for deep lesions that were left in contact with the oral environment for a long time [21]. For such cases, management should include both nerve desensitization as well as occluding the dentinal tubules, possibly by restorations [22]. Femiano et al. investigated the effects of the use of diode laser before composite restoration on symptomatic NCCLs that did not respond to desensitization agents. They found that sensitivity was decreased when the teeth were treated with diode laser prior to restoration [21].

Yet, combining the use of laser desensitizing and restorative treatment for managing NCCL associated with dentin hypersensitivity has little been investigated. Further studies were required to validate this treatment modality in view of the different restorative materials available for treatment of such lesions.

Thus, the aim of this study was to evaluate the influence of diode laser used in the treatment of dentin hypersensitivity on microleakage of NCCL restorations restored with different materials. The null hypotheses were as follows: (1) the use of diode laser for the treatment of dentin hypersensitivity will not increase the risk of microleakage of subsequent cervical restorations; (2) microleakage will not differ among the tested restorative materials, whether with or without the use of diode laser pretreatment; and (3) there will be no difference in microleakage between the occlusal and cervical margins of the restorations, for any of the restorative materials tested and whether with or without the use of diode laser pretreatment.

## 2. Materials and Methods

This in vitro study was reviewed and approved by the research and ethics committee of Dar Al Uloom University, Riyadh, Saudi Arabia (approval # COD/IRB/2020/1). The materials used in the study and their composition, application method, and manufacturers are shown in Table 1.

### 2.1. Tooth Selection

Forty-eight extracted human premolars extracted for orthodontic reasons were collected from pooled unidentified teeth from the Oral Surgery Department of the College of Dentistry at Dar Al Uloom University. The teeth were examined visually and by using an optical microscope at 40x magnification to ensure that they are sound and free from defects. If any cracks, fracture lines, tooth substance loss attrition, or signs of fluorosis, pervious pulpal pathology, or traumatic occlusion were detected, the teeth were excluded from the study. Any calculus deposits and soft tissue were cleaned from the selected teeth using a hand scaler and dental prophylactic cups run at low speed with water-pumice slurry. Teeth were sterilized in an autoclave at 121°C for 20 minutes at 15 lbs psi and then kept in distilled water until use for a maximum of 10 days [23].

### 2.2. Tooth Preparation

**Figure 1:**
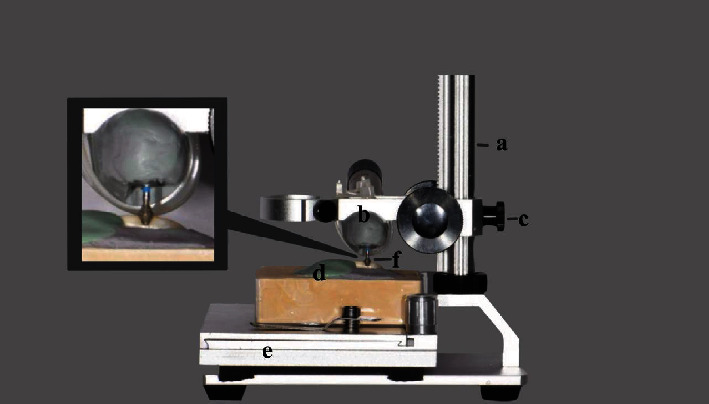
Standardization of tooth preparation using a specially configured assembly with a gliding table and a vertical pole holding a gliding horizontal arm: a—vertical pole; b—horizontal arm holding handpiece; c—lateral screw; d—mold of polyvinyl siloxane; e—gliding table; and f—barrel bur.

A total of ninety-six standardized cervical cavities were prepared on the selected teeth. Each tooth received two preparations: one on the buccal surface and one on the palatal surface [24] using a high-speed handpiece (T2 Boost, SN 717857, Dentsply Sirona, Germany) at 400,000 rpm and under water and air spray. To standardize the preparations, the handpiece was attached to the horizontal arm and fixed to a base stand holder's vertical pole. A lateral screw allowed for controlled up and down movement of the handpiece. The teeth were first placed buccal surface up in a specially designed polyvinyl siloxane mold and stabilized to a gliding table that allowed the slide of the specimens in the *X*- (right and left) and *Y*- (forward and backward) axis. A barrel bur (ISO No. 806.314, IQ DENT, NJ, United States) was fixed to the handpiece. This bur allowed for conical-shaped preparation, with divergent walls and a flat bottom. Prior to tooth preparation, the handpiece holding the bur was stabilized in a vertical position that would allow for cavities to be 1.5 mm in depth. The gliding table was also moved, allowing the cavities to be centralized over the cementoenamel junction of the teeth, in a way that the cervical margins of the preparations were placed on root dentin, while the occlusal margins were on occlusal enamel (Figure 1). Once the initial position was adjusted, the bur was run at high speed under air-water spray, and the gliding table was moved on the *X*-axis to allow the cavity preparation in a mesiodistal direction. The shape and dimension of the burs used allowed for the cervico-occlusal width of the preparations to be 3.0 mm. The cavities' dimensions were confirmed using a digital caliper (Mastercraft electronic caliper, Canadian Tire Corporation, Ltd, ON, Canada) to ±0.01 mm. Once the buccal preparations were completed, the teeth were removed and then replaced in the mold but with the palatal surface up. The whole procedure was repeated as described above to allow for cervical preparations on the palatal surfaces of the teeth with the same dimensions. A new bur was used every four preparations.

### 2.3. Group Allocation and Restoration

The teeth preparations were randomly divided into three groups (*n* = 32) according to the assigned restorative materials tested in this study (Table 1). Each group was divided into two subgroups (*n* = 16) depending on whether they were treated with diode laser or not. To avoid influence of physiologic teeth variations on the results, all cavity preparations on the palatal surfaces were treated by diode laser, prior to restorations, while the preparations on the buccal surfaces were not treated by laser.

Subgroup CR-L: diode laser treatment+restoration with a nanohybrid composite resin

Subgroup CR-NL: immediate restoration with nanohybrid composite resin

Subgroup RMGI-L: diode laser treatment+restoration with RMGI

Subgroup RMGI-NL: immediate restoration with RMGI

Subgroup GIC-L: diode laser treatment+restoration with GIC

Subgroup GIC-NL: immediate restoration with GIC

All preparations were then restored with the material as per the assigned group following manufacturers' recommendations as shown in Table 1. Composite resin restorations were performed after selective enamel etching using 37% phosphoric acid and bonding using a universal adhesive (Universal Single Bond, 3 M ESPE). When used, diode laser treatment was conducted using SIROlaser Blue (Sirona Dental Systems, Bensheim, Germany), where the parameters were set to 970 nm in wavelength, 1.0 W in power and the time was set to 60 seconds. During this time, the light guide was moved back and forth over the entire dentine surface of the prepared cavities.

In Groups CR and RMGI, light polymerization of the restorative materials was conducted using an LED light curing unit (Slimax-C, Beyes Dental, Canada). Light intensity was confirmed by a built-in radiometer to be 1200+/−100 mW/cm2. All restorations were lightly finished and polished using abrasive discs. All teeth were prepared and restored by the same operator to reduce experimental variables.

### 2.4. Thermocycling and Microleakage Testing

After completion of the restorations, the materials were allowed to set for 24 hours before testing. All specimens were subjected to 2,000 thermal cycles between 5°C and 55°C with a dwell time of 30 seconds and 10-second transfer time between baths. Nail polish was then applied 1 mm away from the restoration margins. The roots were sealed with sticky wax and immersed in buffered 1% methylene blue dye solution for 24 hours, then rinsed under tap water. In order to facilitate the cutting procedure, the specimens were mounted in self-cure acrylic resin. Each tooth was sectioned longitudinally in a bucco-lingual direction through the center of the tooth using a diamond wheel run at low speed under water cooling. Microleakage assessment was conducted by two calibrated and blinded examiners, under a light microscope at 40x magnification for dye penetration, from both occlusal and cervical margins using the following scores [24]:

Score of 0: no leakage

Score of 1: leakage depth up to one-third of the internal surface

Score of 2: leakage depth up to two-thirds of the internal surface

Score of 3: leakage through the entire lateral surface to the bottom of the filling

### 2.5. Statistical Analysis

Data were analyzed using statistical software (IBM SPSS for Windows, Version 23.0, IBM SPSS, Inc.) and significance was set at *P* value < 0.05. Frequencies, percentages, median and interquartile range (IQR) were calculated for all variables. Comparisons of microleakage between the three study material groups were done using the Kruskal-Wallis test, followed by multiple pairwise comparisons using Bonferroni adjusted significance. Kappa was calculated to assess the interexaminer agreement when assessing the microleakage scores. Comparisons of microleakage between palatal and buccal restorations (with and without laser desensitization), and occlusal and cervical margins were performed using Wilcoxon signed-rank test. Ordinal logistic regression was used to assess the association of microleakage with material, laser use and margin location. Odds ratio (OR) and 95% confidence interval (CI) were calculated.

## 3. Results

**Figure 2:**
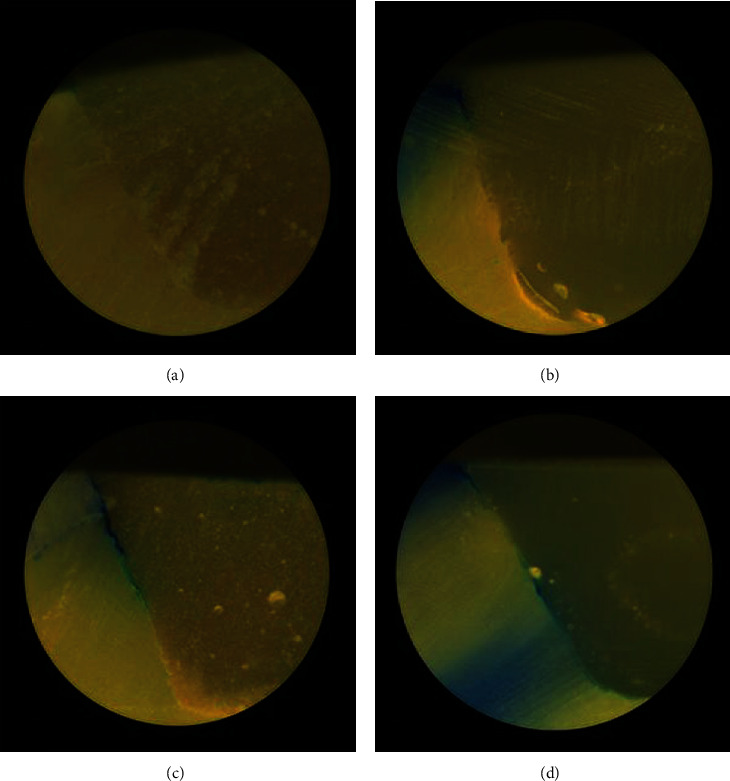
Sample specimens demonstrating different dye penetration scores: (a) occlusal margin of a specimen from subgroup CR-NL showing score 0; (b) cervical margin of a specimen from subgroup RMGI-L showing score 1; (c) occlusal margin of a specimen from subgroup RMGI-L showing score 2; (d) occlusal margin of a specimen from subgroup GI-L showing score 3.

Measurement of interexaminer agreement evaluated by the kappa score ranged from 0.87 to 0.99, indicating excellent reliability. Sample specimens with different dye penetration scores are shown in Figure 2.

The paired *t*-test indicated that there was no statistically significant difference in microleakage scores with the use of laser desensitization, prior to restorative treatment (subgroups RC-L, RMGI-L, and GIC-L) compared to without (subgroups RC-NL, RMGI-NL, and GIC-NL). This was true for all of the three restorative materials tested and at both the occlusal and cervical margins of the restorations (Table 2).

There was a statistically significant difference in the microleakage scores between the different groups of restorative materials tested, whether with or without laser desensitization. The CR group showed the least microleakage, followed by the RMGI group, while the GIC group showed the highest (Table 3).

In general, the cervical margins of the restorations showed greater microleakage scores than the occlusal margins. The difference was statistically significant in both the CR and the RMGI groups with and without laser pretreatment (Table 3).

Regression analysis indicated that the laser treatment had no significant effect on the microleakage scores, while the effect of the material type and the margin location was highly significant (*P* ≤ 0.001) (Table 4).

## 4. Discussion

The increased awareness to preserve natural dentition has amplified the prevalence of NCCLs that are often associated with DH. Although DH, mainly elicited by the hydrodynamic mechanism, is expected to subside after blocking the opened dentinal tubules, the symptoms sometimes persist even after restoring the cervical defects, suggesting that mechanisms other than the hydrodynamic one may contribute to nerve activation. Ladalardo et al. [18] indicated that open dentinal tubules could allow the activation of nerve endings at the dentin-pulpal boundaries, causing the release of neuropeptides and thus inducing neurogenic inflammation. Different treatment modalities are available for dentin desensitization, of which laser treatment first introduced in 1985 by Matsumoto has proven its effectiveness [25]. In particular, low-power lasers that act on the nerve transmission level, rather than altering surface dentin, have recently been gaining popularity.

The immediate reduction of DH reported with the low-power laser treatment may be explained by the experimental concept suggesting that light allows greater passage of calcium, sodium, and potassium ions into the nerve cells. The accumulation of these ions consequently increases the endorphin system and the action potential of the cells, blocking the C fibers afferents, and thus preventing pain information from reaching the central nervous system. In addition to their presumed effectiveness, lower-power diode lasers are now available in the market with reasonable costs, offering an effective treatment of DH, that is less time consuming than other methods in terms of isolation and time of application [26].

Yet, since restoration of NCCL may be required after laser dentin desensitization, validation of this treatment modality needs to be confirmed. In contrast to Nd-YAG laser that causes alterations of the dentinal surface with resultant reduction of the bond strength of the subsequent resin restoration, diode laser mainly acts on the nerve level [27]. However, according to Uman et al., part of the energy could be absorbed by the mineral components of dentin, resulting in the disruption of the crystalline structure, and possibly affecting on the bond strength of the subsequent restoration [28].

Akarsu et al. evaluated the effect of the diode laser used for dentin sensitivity on the clinical success of NCCLs restorations, restored with different adhesive systems [21]. They concluded that diode laser application, prior to the restoration of teeth with NCCL, has reduced hypersensitivity without affecting their retention rates. This finding needed to be confirmed by laboratory investigations; hence the aim of our study was to evaluate the influence of diode laser, used in treatment of dentin hypersensitivity on microleakage of NCCL restorations restored with different materials.

In this study, diode laser with 970 nm wavelength was applied to the dentinal surface for 60 seconds, as these parameters were shown to yield greater therapeutic effects [29–31]. Continuous wave mode, rather than the pulsed mode, was used allowing easier scanning of the whole dentinal surface [30].

Microleakage testing was used to evaluate the influence of diode laser treatment on the success of the restorations. Microleakage, or micro-gaps between the restoration and the cavity walls is an indication of poor adhesion allowing the ingress of fluids, ions and bacteria, leading to post-operative sensitivity and secondary caries and the ultimate failure of the restoration [32]. Different methods are available for microleakage assessment, each having its advantages and disadvantages. In the current study, methylene blue dye was selected among the various techniques because the dye can easily diffuse through the tooth restoration interface, is easily detectable and is a widely used method for microleakage detection [32, 33]. Prior to testing, the restored teeth were subjected to artificial aging using thermocycling. This method simulates the temperature changes that occur in the oral cavity and is an alternative for the more time consuming and costly nature of the clinical studies, especially when the inner structure of the teeth need to be observed [34]. The restored teeth were subjected to 2000 cycles simulating more than 2 months of clinical performance [35].

The results of our study indicated that for all the restorative materials tested, the use of diode laser prior to restoring the noncarious cervical teeth defects, did not significantly affect the microleakage of restorations, necessitating the acceptance of the first null hypothesis. This is in accordance with the clinical findings of Akarsu et al. who found that diode laser did not significantly affect the retention rate, the marginal discoloration, the marginal integrity, nor the secondary caries associated with the restorations of NCCLs [21]. Our findings further highlight the different effect low-power laser such as diode has on the dentinal surface compared to medium-power laser such as Nd:YAG. Although ultrastructural changes to the dentinal surface were shown to occur after diode laser treatment due to some absorption of the laser light by the mineral content of the dentin, these changes, if they had occurred, did not significantly affect the results of our study [28].

Among the many factors affecting microleakage is the type of restorative material and its application method [24, 34]. Composite resin, RMGI and conventional GIC were compared in this study as they are the commonly used esthetic restorative materials indicated for restoring NCCLs.

In this study, whether with or without the use of laser pretreatment, the CR group showed the least microleakage, followed by the RMGI group, while the GIC group showed the highest. This necessitates the rejection of the second null hypothesis. This finding may be attributed to the adhesive method employed when the teeth were restored with CR, where selective enamel etching followed by the application of a self-etching universal adhesive was performed. This method is currently the most recommended as it was shown to improve retention, avoid marginal discoloration and improve longevity of Class V CR restorations [36]. The self-etch adhesive containing 10 methacryloyloxydecyl dihydrogen phosphate (10-MDP), in turn, allows for the creation of a bridge between the hydrophilic tooth substrate and the hydrophobic restoration, allowing for more stable bond strengths and less microleakage [37]. The fact that the use of diode laser treatment prior to CR restoration did not affect the microleakage of the restorations, reassures that this adopted laser treatment modality did not negatively affect the function of the adhesive used nor that of the bonding process. This is in contrast with the findings related to the effect of medium-power laser on microleakage of cervical restorations. Moslemi et al. reported that when Er,Cr:YSGG laser was used, microleakage of cervical restorations was significantly increased [38].

Following manufacturers' recommendations, no primer was used in this study prior to the application of the conventional GIC or RMGI restorations. This may explain the greater microleakage scores noted in groups GIC and RMGI. The function of the primer is to facilitate adhesion of the material to the tooth structure, by modifying the smear layer and allowing for adequate wetting [32].

It was also noted that the conventional GIC showed significantly higher microleakage compared to RMGI. The reported greater solubility in water of the conventional GIC (0.07%) compared to that of RMGI (0.03%), along with its higher moisture sensitivity and lower bond strength to dentin, are reasonable explanations for the significant difference in microleakage scores [39, 40].

In contrast to our findings, Bezera et al. found that GIC showed greater clinical performance and longevity compared to CR after conducting a systemic review and meta-analysis [8]. They included data from randomized and controlled as well as nonrandomized clinical trials, comparing GIC to CR for the restoration of NCCLs. They attributed their finding to the different adhesion mechanisms of the two materials, the difficulty in moisture control in cervical regions of the teeth and the presence of sclerotic dentin, all favoring retention rate and longevity of GIC.

**Figure 3:**
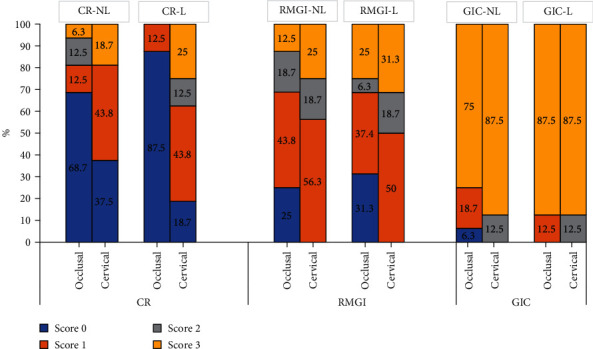
Percentage of microleakage scores associated with the different restorative materials tested with and without diode laser pretreatment: subgroup CR-NL: immediate restoration with nanohybrid composite resin; subgroup CR-L: diode laser treatment+restoration with a nanohybrid composite resin; subgroup RMGI-NL: immediate restoration with RMGI; subgroup RMGI-L: diode laser treatment+RMGI restoration; subgroup GIC-NL: immediate restoration with GIC; and subgroup GIC-L: diode laser treatment+GIC restoration.

The results of the current study also revealed that cervical margins of the restorations in all restorative material groups showed greater microleakage scores compared to the occlusal margins, necessitating the rejection of the third null hypothesis. The difference was not statistically significant in the GIC group with or without the laser pretreatment, and this is because the microleakage score at the occlusal margins were already high, concealing the difference compared to the gingival margins. As in agreement with numerous studies [24, 32, 36], adhesion at the cervical margins of the restorations is less reliable than to the occlusal margins. Several factors contribute to this finding, including the lower mineral content of dentin compared to enamel and its more complex structural pattern. Nevertheless, and in agreement with other studies [41], it is important to mention that all restorative material tested in this study showed some degree of microleakage that varied according to the restorative material used and to the margin location, as shown in Figure 3.

The most important limitation of this study is the type of dentin to which the restorative procedures were conducted. In the current study, cavities were artificially prepared at the cervical region of the teeth, rather than obtaining extracted teeth that have NCCLs naturally. NCCLs usually exhibit a high degree of dental sclerosis, which can hinder adequate conditioning prior to composite resin restorations, while on the other hand, they could favor bonding with materials that establish chemical bonds with the dentin's mineral substrate, such as GIC and RMGI [8]. Another limitation is the in vitro nature of the study, which does not put into consideration the difficulty of obtaining adequate moisture control when restoring NCCLs—another factor that may have favored GIC and RMGI [42]. Thus, even though the results of our study reassure that diode laser for the treatment of dentin hypersensitivity will not affect the microleakage of NCCL restorations, further clinical and laboratory studies will be recommended to confirm the safety and validity of this treatment approach.

## 5. Conclusion

Within the limitations of the study, the following can be concluded:

1. Application of diode laser for the treatment of DH prior to the restoration of teeth with NCCL will not affect the microleakage of the all the tested restorative materials.
2. All the materials used showed some degree of microleakage which was higher in gingival margins compared to occlusal margins.
3. The resin composite showed the least microleakage compared to resin-modified glass ionomer and glass ionomer restorations.

## Figures and Tables

**Table 1:** The materials used in the study and their composition, application method, and manufacturers.

Materials (class of material—group code)	Compositions^∗^	Method of application	Manufacturer
3M Universal Single Bond (universal adhesive)	MDP phosphate monomer, dimethacrylate resin, HEMA, ethanol, silane	Applied to the entire surface of the enamel and dentin and rubbed for 20 seconds. Gently air dried for approximately 5 seconds and light cured for 10 sec	3M ESPE, St. Paul, MN, USA

Filtek Z350 XT (nanohybrid composite resin—CR)	Bis-GMA, bis-EMA, UDMA, TEGDMA, zirconia/silica (particle size = 20-75 nm, cluster size = 0.6-1.4 *μ*m, 59.5 vol%)	Placed incrementally after adhesive was applied. Each increment was light cured for 20 sec	3M ESPE, St. Paul, MN, USA

Photac Fil (resin-modified glass ionomer cement—RMGI)	Polyethylene polycarbonic acid 2, hydroxyethyl methacrylate, water, diurethane dimethacrylate, magnesium, HEMA, ester	The capsule was activated and mixed in an amalgamator for 10 sec and placed in two-increment bulk. Each increment was light cured for 20 msec	3M ESPE, St. Paul, MN, USA

Ketac Universal (conventional glass ionomer cement—GIC)	Powder: oxide glass > 95 wt%. Liquid: water (40–60 wt%), copolymer of acrylic acid-maleic acid (30-50 wt%), tartaric acid (1-10 wt), and benzoic acid (<0.2 wt%)	The capsule was activated and mixed in an amalgamator for 10 sec and placed in bulk	3M ESPE Dental Products, St. Paul, USA

MDP phosphate: 10-methacryloyloxydecyl dihydrogen phosphate; HEMA: 2-hydroxyethyl methacrylate; bis-GMA: bisphenol A-glycidyl methacrylate; bis-EMA: ethoxylated bisphenol glycol dimethacrylate; UDMA: urethane dimethacrylate; TEGDMA: triethylene glycol dimethacrylate. ^∗^Compositions are as disclosed by the manufacturers.

**Table 2:** Comparison of microleakage scores according to diode laser pretreatment for the three restorative material groups tested.

			Subgroup NL	Subgroup L	WSR
					*P* value
Group CR (Filtek Z350 XT)	Occlusal margin	Score 0	11 (68.8%)	14 (87.5%)	0.08
		Score 1	2 (12.5%)	2 (12.5%)	
		Score 2	2 (12.5%)	0 (0%)	
		Score 3	1 (6.3%)	0 (0%)	
		Median (IQR)	0.00 (1.00)	0.00 (0.00)	
	Cervical margin	Score 0	6 (37.5%)	3 (18.8%)	0.26
		Score 1	7 (43.8%)	7 (43.8%)	
		Score 2	0 (0%)	2 (12.5%)	
		Score 3	3 (18.8%)	4 (25%)	
		Median (IQR)	1.00 (1.00)	1.00 (1.75)	

Group RMGI (Photac Fil)	Occlusal margin	Score 0	4 (25%)	5 (31.3%)	0.73
		Score 1	7 (43.8%)	6 (37.5%)	
		Score 2	3 (18.8%)	1 (6.3%)	
		Score 3	2 (12.5%)	4 (25%)	
		Median (IQR)	1.00 (1.75)	1.00 (2.75)	
	Cervical margin	Score 0	0 (0%)	0 (0%)	0.32
		Score 1	9 (56.3%)	8 (50%)	
		Score 2	3 (18.8%)	3 (18.8%)	
		Score 3	4 (25%)	5 (31.3%)	
		Median (IQR)	1.00 (1.75)	1.00 (2.00)	

Group GIC (Ketac Universal)	Occlusal margin	Score 0	1 (6.3%)	0 (0%)	0.18
		Score 1	3 (18.8%)	2 (12.5%)	
		Score 2	0 (0%)	0 (0%)	
		Score 3	12 (75%)	14 (87.5%)	
		Median (IQR)	3.00 (1.50)	3.00 (0.00)	
	Cervical margin	Score 0	0 (0%)	0 (0%)	1.00
		Score 1	0 (0%)	0 (0%)	
		Score 2	2 (12.5%)	2 (12.5%)	
		Score 3	14 (87.5%)	14 (87.5%)	
		Median (IQR)	3.00 (0.00)	3.00 (0.00)	

WSR: Wilcoxon signed-rank test; NL: without laser pretreatment; L: with laser pretreatment.

**Table 3:** Comparison of microleakage scores in the three study groups with and without laser treatment.

	Margin	Score	Group CR (Filtek Z350 XT)	Group RMGI (Photac Fil)	Group GIC (Ketac Universal)	KWT
						*P* value
			*N* (%)			
Subgroup NL	Occlusal margin	Score 0	11 (68.8%)	4 (25%)	1 (6.3%)	<0.001^∗^
		Score 1	2 (12.5%)	7 (43.8%)	3 (18.8%)	
		Score 2	2 (12.5%)	3 (18.8%)	0 (0%)	
		Score 3	1 (6.3%)	2 (12.5%)	12 (75%)	
		Median (IQR)	0.00 (1.00)^a^	1.00 (1.75)^a^	3.00 (1.50)^b^	
	Cervical margin	Score 0	6 (37.5%)	0 (0%)	0 (0%)	<0.001^∗^
		Score 1	7 (43.8%)	9 (56.3%)	0 (0%)	
		Score 2	0 (0%)	3 (18.8%)	2 (12.5%)	
		Score 3	3 (18.8%)	4 (25%)	14 (87.5%)	
		Median (IQR)	1.00 (1.00)^a^	1.00 (1.75)^a^	3.00 (0.00)^b^	
		WSR *P* value	0.008^∗^	0.01^∗^	0.10	
Subgroup L	Occlusal margin	Score 0	14 (87.5%)	5 (31.3%)	0 (0%)	<0.001^∗^
		Score 1	2 (12.5%)	6 (37.5%)	2 (12.5%)	
		Score 2	0 (0%)	1 (6.3%)	0 (0%)	
		Score 3	0 (0%)	4 (25%)	14 (87.5%)	
		Median (IQR)	0.00 (0.00)^a^	1.00 (2.75)^b^	3.00 (0.00)^c^	
	Cervical margin	Score 0	3 (18.8%)	0 (0%)	0 (0%)	<0.001^∗^
		Score 1	7 (43.8%)	8 (50%)	0 (0%)	
		Score 2	2 (12.5%)	3 (18.8%)	2 (12.5%)	
		Score 3	4 (25%)	5 (31.3%)	14 (87.5%)	
		Median (IQR)	1.00 (1.75)^a^	1.00 (2.00)^a^	3.00 (0.00)^b^	
		WSR *P* value	0.001^∗^	0.03^∗^	0.16	

KWT: Kruskal-Wallis test; WSR: Wilcoxon signed-rank test; NL: without laser pretreatment; L: with laser pretreatment. ^∗^Statistically significant at *P* value < 0.05. ^a, b, c^Different letters denote statistically significant differences between groups using the Bonferroni adjustment for multiple pairwise comparisons.

**Table 4:** Association of different factors with microleakage scores.

		Unadjusted model		Adjusted model	
		OR (95% CI)	*P* value	OR (95% CI)	*P* value
Restorative material	Group CR (Filtek Z350 XT)	0.02 (0.006, 0.04)	<0.001^∗^	0.01 (0.005, 0.03)	<0.001^∗^
	Group RMGI (Photac Fil)	0.07 (0.03, 0.16)	<0.001^∗^	0.06 (0.02, 0.13)	<0.001^∗^
	Group GIC (Ketac Universal)	Reference category			
Laser pretreatment	With	1.13 (0.68, 1.90)	0.64	1.18 (0.65, 2.13)	0.59
	Without	Reference category			
Margin	Occlusal	0.41 (0.24, 0.70)	0.001^∗^	0.26 (0.14, 0.49)	<0.001^∗^
	Cervical	Reference category			

Model *X*^2^: 73.78. ^∗^*P* value < 0.001. OR: odds ratio; CI: confidence interval. ^∗^Statistically significant at *P* value < 0.05.

## Data Availability

The microleakage data used to support the findings of this study are available from the corresponding author upon request.
